# Supplementary material for: Thin film epitaxial [111] Co50Pt50: structure, magnetisation, and spin polarisation
Source: Sci Rep. 2023 Aug 1;13:12468. doi: 10.1038/s41598-023-37825-3 (PMC10394051; doi:10.1038/s41598-023-37825-3)
Supplement: Supplementary file 1 — Supplementary Information. [file 41598_2023_37825_MOESM1_ESM.pdf]

Supplementary Information for "Thin film epitaxial [111] $Co_{50}Pt_{50}$: Structure, magnetisation, and spin polarisation"

N. Satchell[1], S. Gupta[1], M. Maheshwari[1], P. M. Shepley[1], M. Rogers[1], O. Cespedes[1], and G. Burnell[1,*]

[1]School of Physics and Astronomy, University of Leeds, Leeds, LS2 9JT, United Kingdom
[*]g.burnell@leeds.ac.uk

ABSTRACT

This Supplementary Information contains additional analysis and data in support of the conclusions presented in the main text.

X-ray Diffraction Data

Figures S1, S2, S3 show a complete set of X-ray diffraction scans for samples grown at each temperature. The very sharp peak at $2\theta \approx 42^\circ$ is from the c-plane sapphire $(0\,0\,0\,6)$ plane with $\{1\,1\,1\}$ peaks from the CoPt phase clearly visible to left. The substrate $(0\,0\,0\,12)$ and CoPt $\{2\,2\,2\}$ peaks are also present close to 2θ of 90°. Samples grown at $200-450^\circ$C and also at 800°C clearly exhibit Pendellosung fringes, indicative of good crystal ordering from the $L1_1$ and $L1_0$ phases. The peak intensity and FWHM shown in the main text are extracted from a Gaussian peak fit to the CoPt $\{1\,1\,1\}$ peak data, although similar data could be obtained from the CoPt $\{2\,2\,2\}$ data. Figure S4 shows the XRD on the 40 nm and 128 nm samples grown at 350°C. The additional peak at $\approx 21^\circ$ in the 128 nm sample we interpret as the superstructure peak from the $L1_1$ growth.

X-ray Reflectivity Data

Growth Temp	Al_2O_3	Pt1			CoPt			Pt2		
(°C)	σ (Å)	d (Å)	σ (Å)	ρ (f.u./Å^3)	d (Å)	σ (Å)	ρ (f.u./Å^3)	d (Å)	σ (Å)	ρ (f.u./Å^3)
27	4.9	42.8	5.6	0.0615	385	9.6	0.0758	40.9	8.0	0.0713
200	3.7	44.8	3.3	0.0615	382	10.8	0.0758	39.0	6.2	0.0713
250	3.3	46.0	3.9	0.0615	381	9.7	0.0758	40.8	5.8	0.0713
300	3.2	43.2	3.8	0.0615	388	13.5	0.0758	37.2	5.7	0.0713
350	2.8	42.1	4.1	0.0615	387	16.9	0.0758	36.4	4.8	0.0713
400	2.6	41.5	4.6	0.0615	388	15.7	0.0758	39.4	5.9	0.0713
450	3.3	41.1	4.1	0.0615	395	20.7	0.0758	32.2	7.5	0.0713
550	4.6				445	34.0	0.0758	29.6	8.9	0.0641
650	5.0				477	14.0	0.0812			
700	5.4	7.6	4.2	0.0615	385	54.5	0.0758	61.6	19.1	0.0564
750	4.4				481	10.0	0.0786			
800	5.4				479	9.5	0.0796			
850	5.2				493	13.0	0.0724			

Figures S5, S6, S7 show a complete set of X-ray reflectivity scans for samples grown at each temperature. The data with best fits are shown on the left, with the model corresponding to the best fit on the right side. The XRR are fit using the GenX package[1]. The extracted fitting parameters are given in the following table. Each layer is modelled as a box with a thickness (d) and a roughness (σ), in units of Å, and a density (ρ), in units of formula unit (f.u.)/Å^3. The substrate has only a roughness fitting parameter as the model treats it as in infinitely thick box with the bulk density of Al_2O_3. "Pt1" layer is the seed layer and "Pt2" is the capping layer. Between 27°C and 450°C, the best fit model is for the as-intended trilayer. Between these temperatures the density is fixed and only the thickness and roughness are free fit parameters. The slight difference in density

between the seed and capping layer indicates that the top surface of "Pt2" is oxidized. At higher temperatures the trilayer model breaks down and alternative fitting to either a single, bi-, or tri-layer model is used.

Magnetometry

Magnetic hysteresis loops were measured for a set of samples of common thickness grown at temperatures from room temperature to 850° C with the field applied in the plane of the substrate and perpendicular to the substrate plane - shown in Figure S8. For the data shown here, a diamagnetic background resulting from the substrate and sample stick has been removed. The saturation magnetisation is determine by fitting a line to the outer section of the upper and lower branches and taking the difference in intercepts with the $H = 0$ axis. The uncertainty is dominated by the determination of the sample area. The saturation field is determined from the point at which the measured data has deviated from the saturation magnetisation by more than twice the scatter in the data in the saturated regions of the loop. Figures S9 and S10 show similar magnetic hysteresis loops for samples of increasing thickness grown at 350° C and 800° C respectively.

PCAR

The Point Contact Andreev Reflection spectra are measured by an AC lockin technique where the contact is subjected to a controlled DC bias with a small AC modulation which is sensed across the contact and across a control resistor to allow a differential conductance to be determined. The DC bias is separately monitored as it is adjusted to give a tip bias typically in the range of $\approx \pm 30\,\mathrm{meV}$. The DC bias is swept over 6 quarters from 0 to maximum and then to minimum and back to maximum and finally to zero. We only analyse the parts of the trace from maximum to minimum and back to maximum. The measured DC bias is subject to a small instrumentation offset which is removed at this first stage of data processing. Since all the features of interest in the spectra should be symmetrical about zero bias, we decompose the data into symmetric and anti-symmetric parts and retain only the symmetric part. The BTK model is usually considered in terms of the normalised conductance of the contact – normalised to the conductance at high bias where transfer between electron/hole states in the ferromagnetic and electron/hole-like quasiparticles in the superconductor. In this high bias regime it is possible in some contact to get parabolic conductance due to tunnelling or Joule heating effects[2] and so we normalise the experimental data by dividing through a parabola fitted to the outer 20% of the spectra.

The modified BTK model used in the analysis of the PCAR data has 4 fitting parameters. Firstly, an interfacial barrier strength Z that is dimensionless. This effectively determines a scattering potential at the interface and so even in a perfect contact will take a small, but finite, value. Secondly, the spin polarisation $P = \frac{N_\uparrow v_{f\uparrow} - N_\downarrow v_{f\downarrow}}{N_\uparrow v_{f\uparrow} + N_\downarrow v_{f\downarrow}}$ which thus ranges from 0 for non-spin-polarised materials to 1 for fully spin-polarised materials. Thirdly, the superconducting energy gap Δ which might reasonably be expected not to be fully bulk like at the tip of the contact. Finally there is a 'smearing parameter' ω that encompasses both thermal effects and athermal scattering processes not otherwise accounted for in the model.

The modified BTK model is then fitted to the now normalised data. We start with an initial set of fitting values of $Z = 0.5$, $\omega = 0.5\,\mathrm{meV}$ (c.f. $4.2\,\mathrm{K} \equiv 0.36\,\mathrm{meV}$), $\Delta = 1.5\,\mathrm{meV}$ and $P = 0.5$. We use a differential-evolution algorithm to identify a global minimum χ^2 value to locate the best fit. This best-fit location is then used to seed a non-linar least squares fit using the Levenberg–Marquardt algorithm, allowing us to determine estimated standard errors for the fitting parameters.

We then discard fits with non-physical values of Δ, excessibley large ω – both of which would indicate an unaccounted spreading resistance, before considering all the fitted (Z, P) combinations. As noted in the main text, the extrapolation back to $Z^2 = 0$ is not strictly physical, however, in practice in an all-metal system, very low values of Z with uncertainties that encompass $Z = 0$ can be obtained.

References

1. Björck, M. & Andersson, G. *GenX*: an extensible X-ray reflectivity refinement program utilizing differential evolution. *J. Appl. Crystallogr.* **40**, 1174–1178, DOI: 10.1107/S0021889807045086 (2007).
2. Baltz, V. *et al.* Conductance features in point contact Andreev reflection spectra. *J. Physics: Condens. Matter* **21**, 095701, DOI: 10.1088/0953-8984/21/9/095701 (2009).

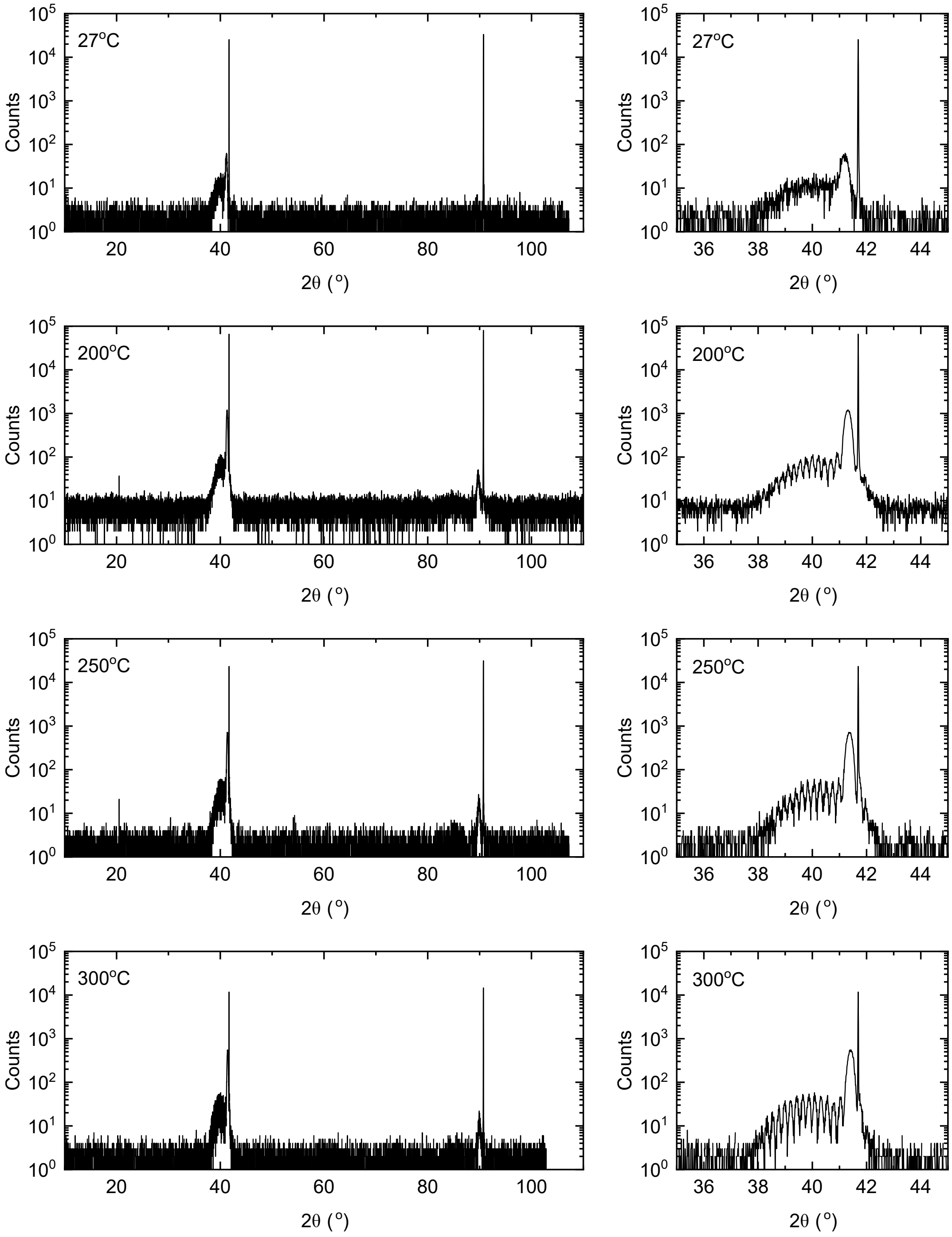

Figure S1. X-ray diffraction for the growth temperature set of samples reported in the main text. Left column: Full data range. Right column: Cropped region around the 111 structural peak.

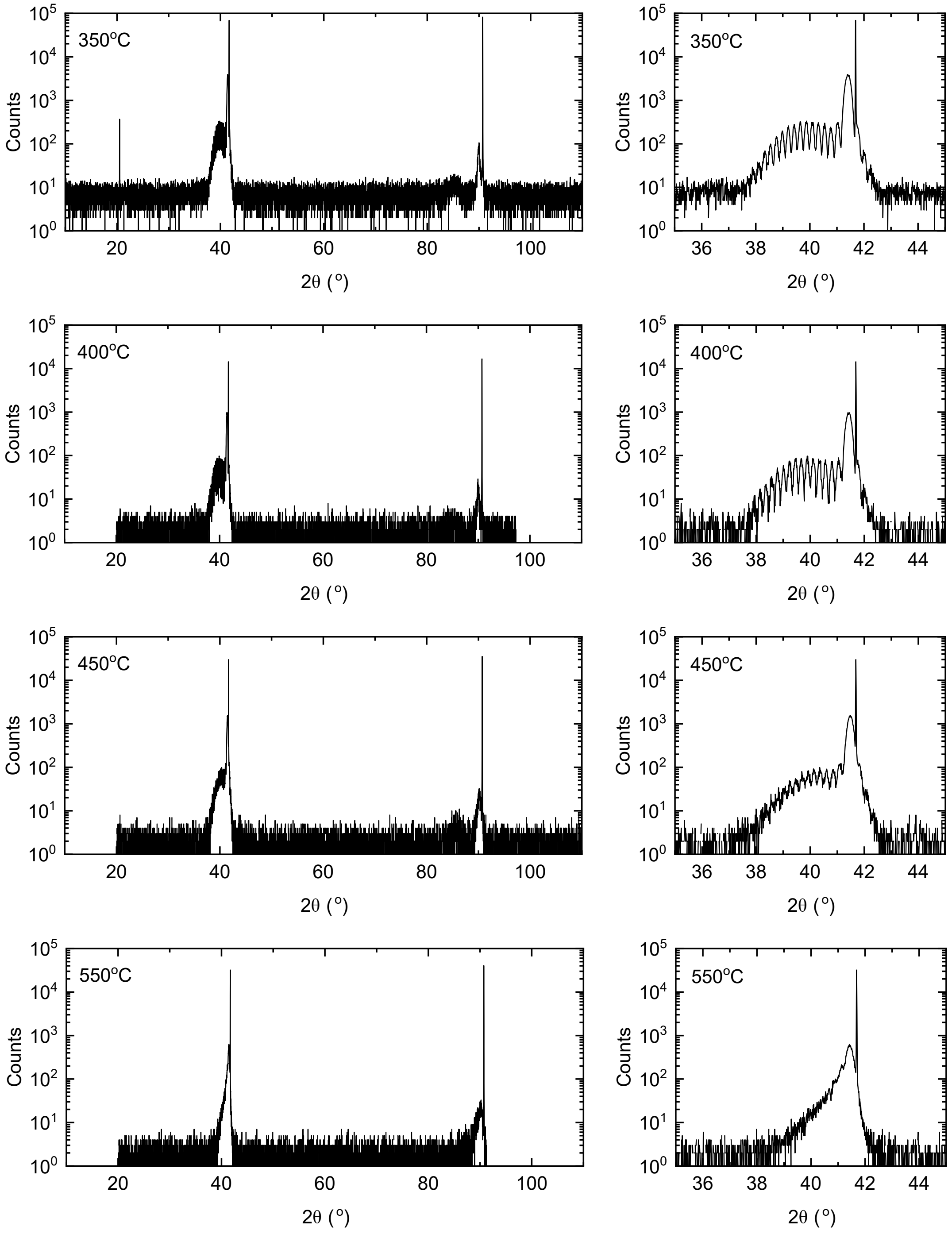

Figure S2. X-ray diffraction for the growth temperature set of samples reported in the main text. Left column: Full data range. Right column: Cropped region around the 111 structural peak.

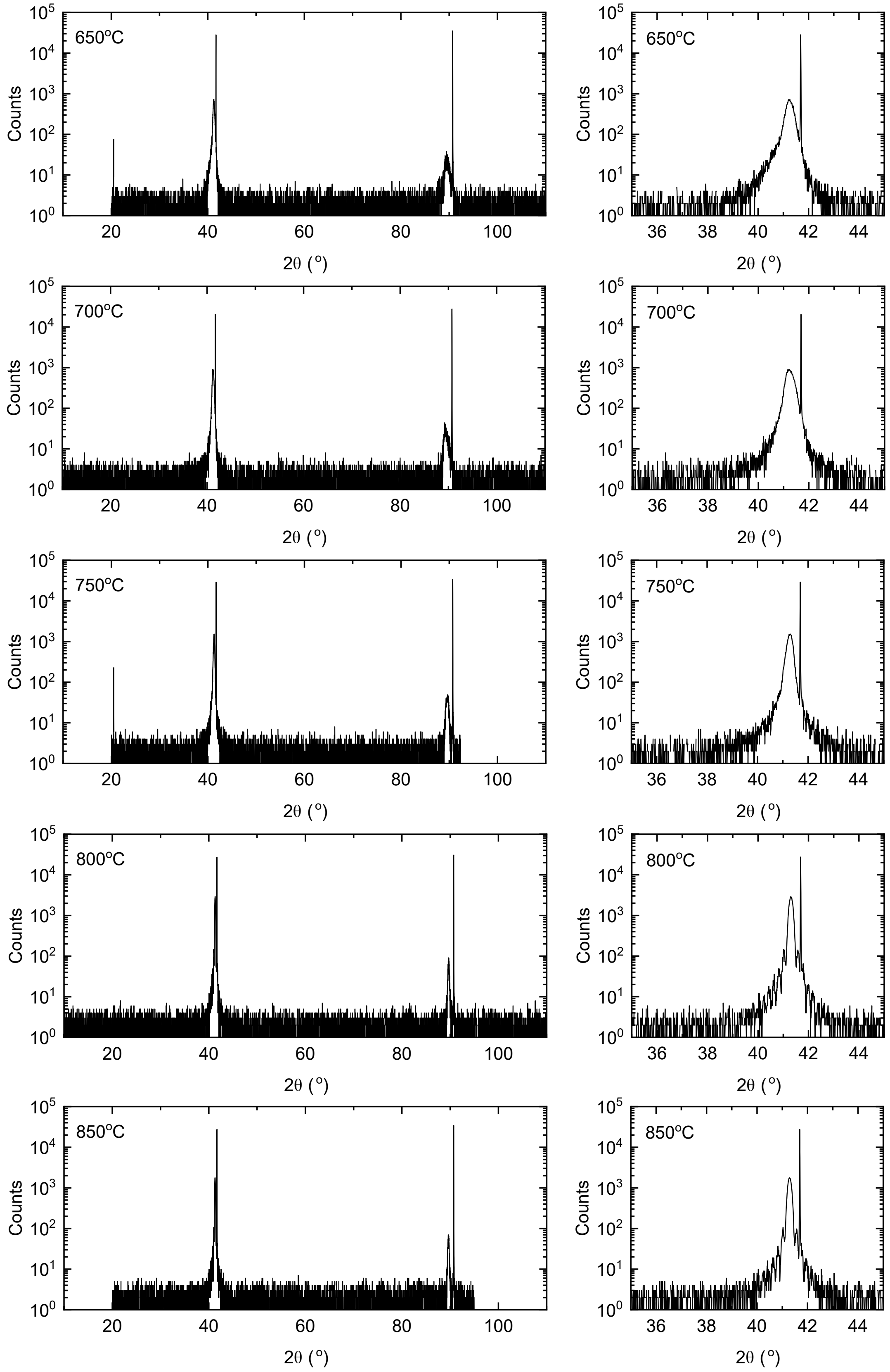

Figure S3. X-ray diffraction for the growth temperature set of samples reported in the main text. Left column: Full data range. Right column: Cropped region around the 111 structural peak.

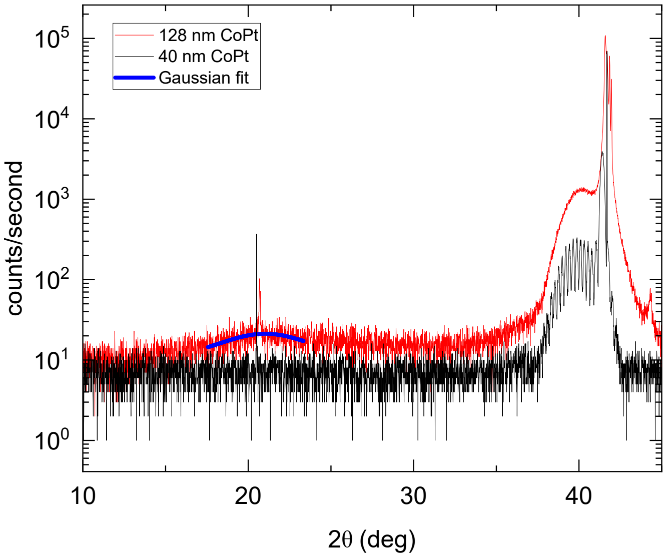

Figure S4. X-ray diffraction for the 350°C growth temperature over selected range. The 128 nm sample shows evidence of a superstructure peak at $2\theta \approx 21°$.

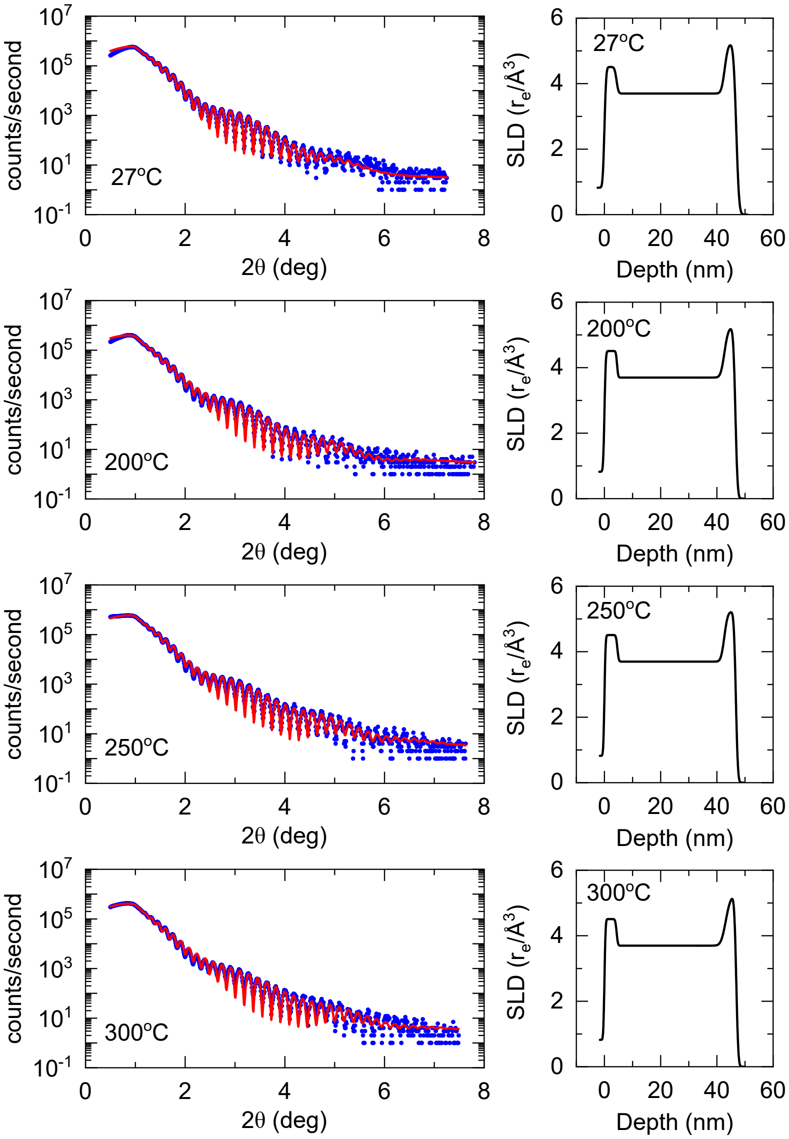

Figure S5. X-ray reflectivity for the growth temperature set of samples reported in the main text. Left column: Full data range with best fit line. Right column: Structural model corresponding to best fit.

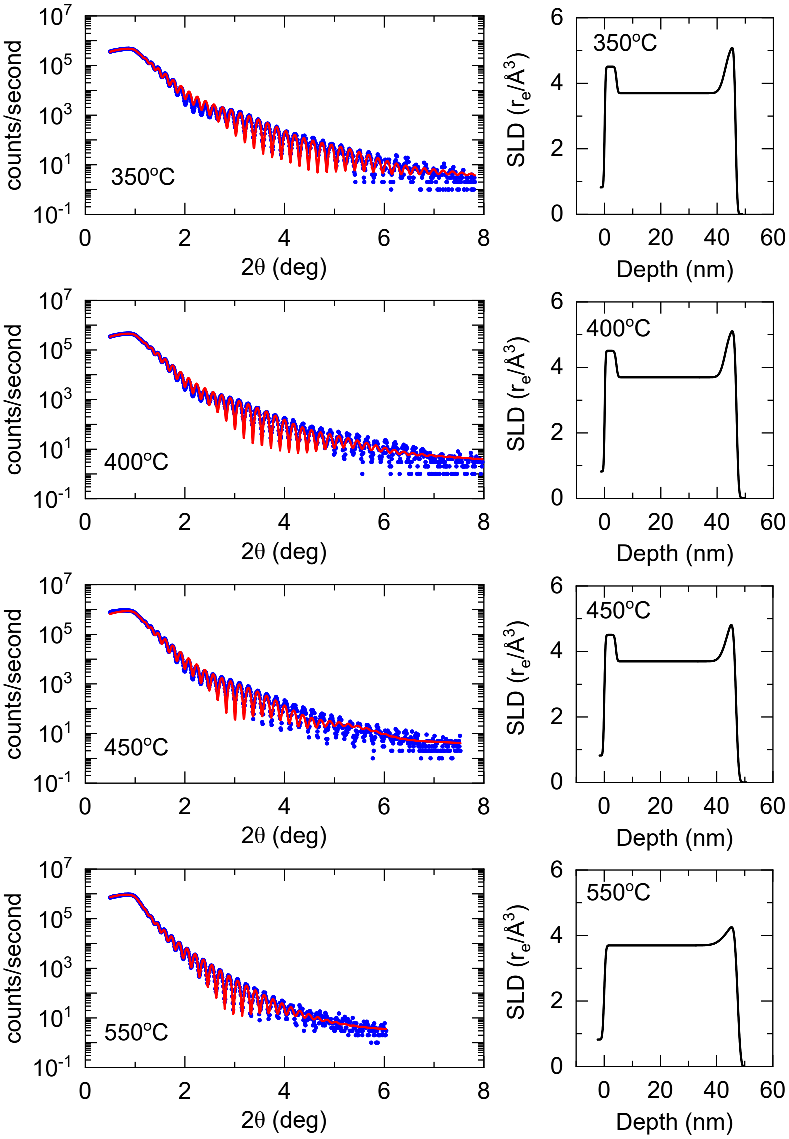

Figure S6. X-ray reflectivity for the growth temperature set of samples reported in the main text. Left column: Full data range with best fit line. Right column: Structural model corresponding to best fit.

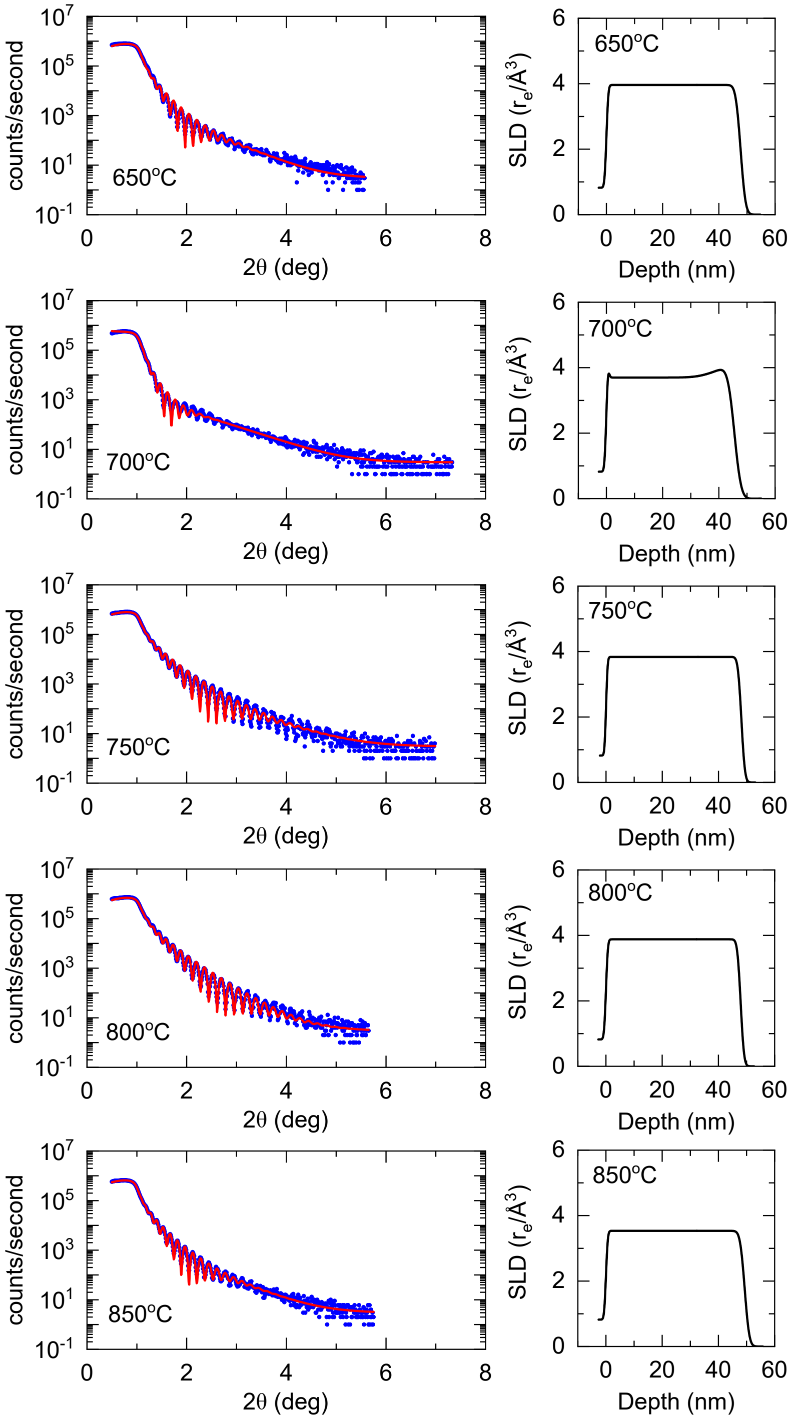

Figure S7. X-ray reflectivity for the growth temperature set of samples reported in the main text. Left column: Full data range with best fit line. Right column: Structural model corresponding to best fit.

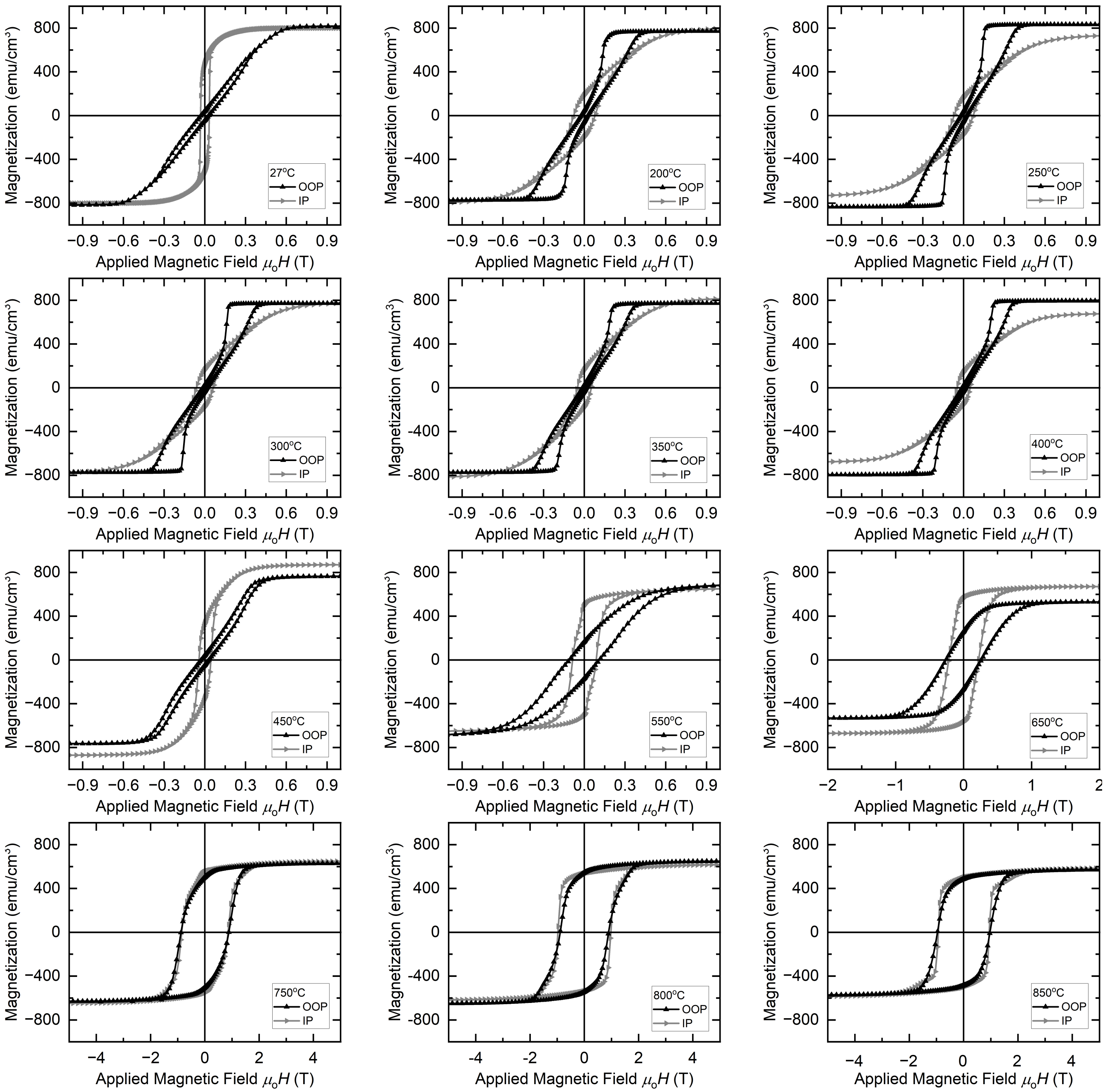

Figure S8. Volume magnetisation for the growth temperature set of samples reported in the main text. The thickness of all samples is 40 nm.

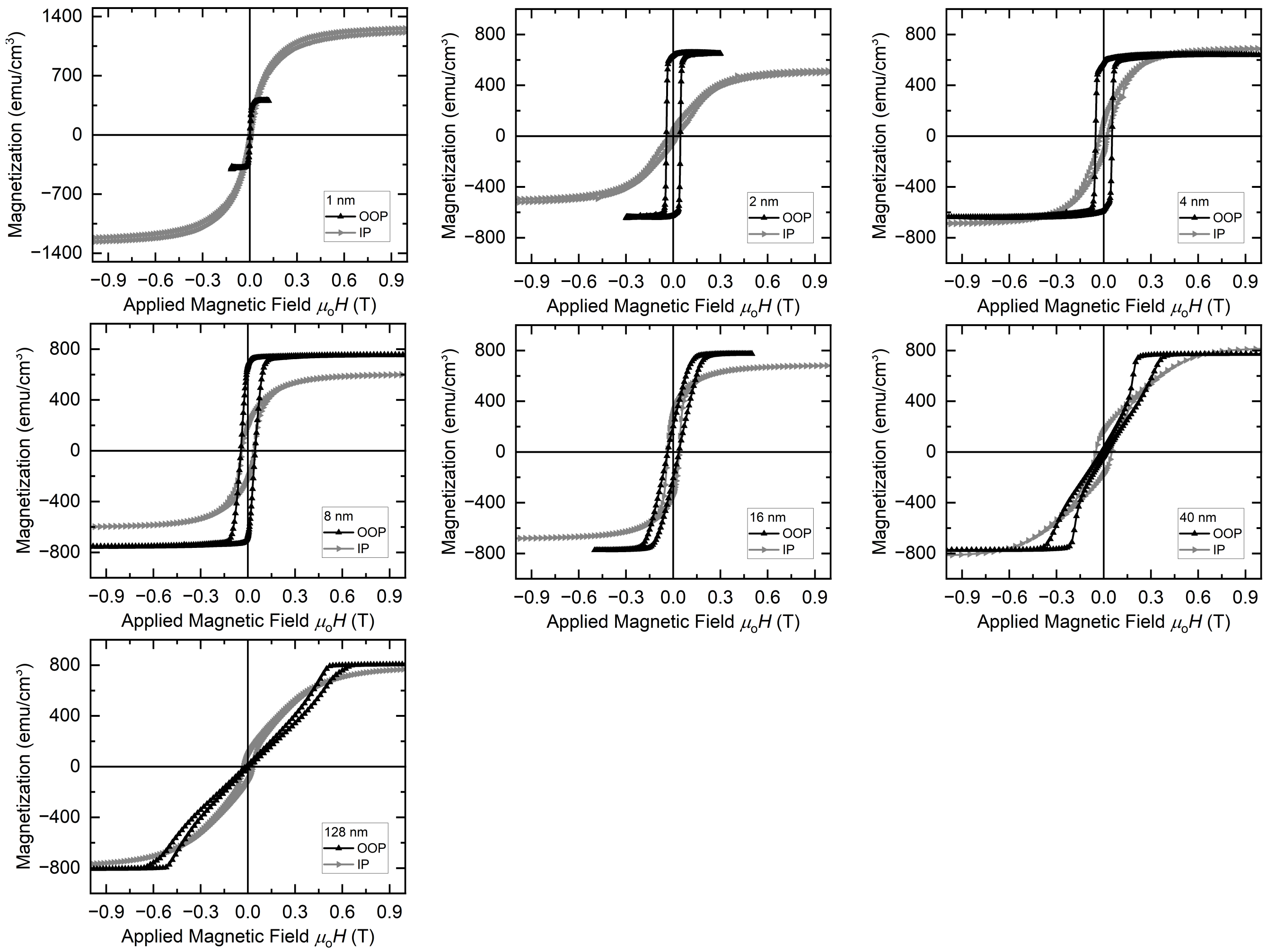

Figure S9. Volume magnetisation for the thickness series set of samples reported in the main text for growth at 350°C.

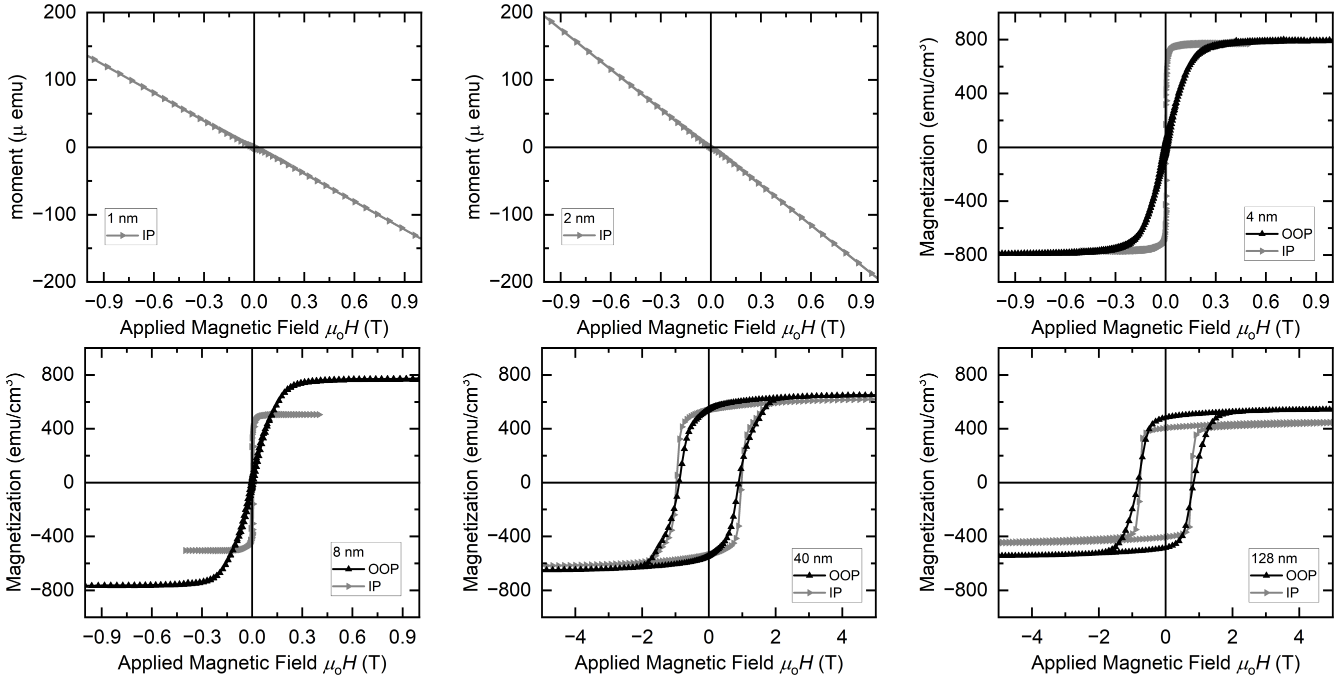

Figure S10. Volume magnetisation for the thickness series set of samples reported in the main text for growth at 800°C. For the 1 nm and 2 nm samples which did not show ferromagnetism, the uncorrected moment vs field is presented.
